# Inflammatory and deleterious role of gut microbiota-derived trimethylamine on colon cells

**DOI:** 10.3389/fimmu.2022.1101429

**Published:** 2023-01-16

**Authors:** Rekha Jalandra, Govind K. Makharia, Minakshi Sharma, Anil Kumar

**Affiliations:** ^1^ Gene Regulation Laboratory, National Institute of Immunology, New Delhi, India; ^2^ Department of Zoology, Maharshi Dayanand University, Rohtak, India; ^3^ Department of Gastroenterology and Human Nutrition, All India Institute of Medical Sciences, New Delhi, India

**Keywords:** metabolite, TMA, genotoxic, cytotoxic, colorectal cancer, trimethylamine, inflammatory, deleterious

## Abstract

Trimethylamine (TMA) is produced by the intestinal microbiota as a by-product of metabolism of dietary precursors. TMA has been implicated in various chronic health conditions. However, the effect of TMA in the colon and the underlying mechanism was not clear. In this study, TMA exhibited toxic effects *in vitro* as well as *in vivo*. TMA-induced oxidative stress causes DNA damage, and compromised cell membrane integrity leading to the release of LDH outside the cells which ultimately leads to cell death. Besides, TMA also exhibited pronounced increase in cell cycle arrest at G2/M phase in both HCT116 and HT29 cell lines. TMA was found to be genotoxic and cytotoxic as the TMA concentration increased from 0.15 mM. A decreased ATP intracellular content was observed after 24 h, 48 h, and 72 h treatment in a time and dose-dependent manner. For *in vivo* research, TMA (100 mM, i.p. and intra-rectal) once a week for 12 weeks caused significant changes in cellular morphology of colon and rectum epithelium as assessed by H & E staining. TMA also significantly increased the infiltration of inflammatory cells in the colon and rectal epithelium indicating the severity of inflammation. In addition, TMA caused extensive mucosal damage and distortion in the epithelium, decrease in length of small intestine compared to control mice. In conclusion, these results highlight the detrimental effects of TMA in the colon and rectal epithelium.

## Introduction

The digestion of dietary components such as choline, carnitine, and betaine by gut microbiota leads to the production of trimethylamine (TMA), an amine compound (1). The anaerobic conversion of dietary sources to TMA occurs mostly in the caecum and colon. Notably, Gammaproteobacteria, Betaproteobacteria, Firmicutes, and Actinobacteria can synthesize TMA (2, 3). Mostly, TMA gets passively diffuses into blood circulation through the enterocyte membrane. TMA is transported to the liver where it is converted into Trimethylamine-N-Oxide (TMAO) by hepatic FMO1 and FMO3 enzymes (4–6). TMAO is subsequently excreted through urine. The ratio of excreted TMA: TMAO is 3:95 and 95% of TMA gets oxidized and excreted. The reported amount of TMA in the fecal sample of healthy volunteers is approximately 0.146-0.5 μmol/g (7, 8). Only 4% of TMA is excreted in feces (3, 9). Another study denies the excretion of TMA in feces which may be due to the sensitivity of the detection method (10). Therefore, the level of TMA in feces seems lacunae in understanding its role. The level of TMA in the body depends on the dietary intake of choline and carnitine (11, 12). An increase in choline-rich diet intake elevates blood TMA and TMAO concentration (13). Therefore, the amount of TMA produced is directly proportional to the precursor and activity of bacteria catalyzing TMA formation. In a urinary metabolomics study of colorectal cancer patients and healthy controls, TMAO was listed as a metabolite that can be selected as a marker to discriminate between colorectal cancer (CRC) patients and healthy individuals (14).

Various studies assessing the role of TMAO reported divergent results depending upon the cell line used but the role of TMA has never been evaluated despite having proven toxic effects. The toxicity and deleterious effects of TMA were first evaluated in 1885 (15–17). Increased level of TMA in the body in fish odor syndrome causes fish-like odor in the body (18). Studies show diversity in terms of concentration of TMA in the urine of healthy individuals, ranging from an average of few μM to 50 mM due to variations in food habits and the sensitivity of instrument used for measurement (19, 20). Some studies reported even lesser concentration of TMA in body fluid (21, 22). However, only a few studies have reported TMA abundance in body fluids such as serum, and plasma in cardiovascular disease but there is a paucity of evidence in colorectal cancer (16, 23, 24). These findings suggest that the TMA is more detrimental in comparison to TMAO. Few facts suggest that TMA is more toxic than TMAO and should have drawn the attention of researchers earlier. First, the liver detoxifies the human body from harmful molecules and in the course, it oxidizes TMA to TMAO suggesting that TMA is more toxic than TMAO. Second, TMAO is a well-known osmolyte that stabilizes protein structure in marine animals (25). Third, TMA has a strong unpleasant odor which is an alarm of toxic food (26). Therefore, this study reports the effect of TMA on adenocarcinoma cell lines (HCT116 and HT29) and mice model. Our research found the acute and long-term effects of TMA on both cell lines with an emphasis on cytotoxic and genotoxic effects. Whereas, the effect of TMA in mice, assessed in terms of histology scores, shows a severe inflammation has been caused by TMA in both the colon and rectum. Additionally, the link between these cellular parameters is also discussed.

## Methods

### Cell culture and proliferation

The human adenocarcinoma cell lines HCT116 (colorectal carcinoma, ATCC ^®^ CCL-247) was purchased from ATCC, and HT29 (colorectal adenocarcinoma, ATCC ^®^ HTB-38) was borrowed from another lab in the same institute. The HT29 cell line was revived within 15 days of receiving it from ATCC and HT29 cell line was authenticated by the lab from which we received it. Cells were grown in Dulbecco’s modified Eagle’s medium-F12 nutrient mixture (DMEM-F12; Gibco) supplemented with 10% heat-inactivated fetal bovine serum (FBS; Gibco) and 100 I.U./ml penicillin and 100 µg/ml streptomycin (Sigma-Aldrich; Merck KGaA, Germany). Cells were allowed to grow at 37°C under 5% CO_2_ in a humified incubator. Cells were cultured in a 96-well plate, 6-well plate with or without TMA freshly prepared in PBS. The culture medium was changed every 24 h. For cell proliferation, cells were treated with different concentrations of TMA from 0.15 mM to 10 mM and controls were without TMA for 24, 48, and 72 h of the incubation period. After treatment cells were recovered using 0.1% trypsin-EDTA. All experiments were performed with cells in the logarithmic phase of growth. The bacterial metabolite, TMA, was purchased from Sigma-Aldrich; Germany.

### Cell viability

To check the cytotoxic potential of bacterial metabolite, commercially available TMA, on epithelial cell line HCT116 and HT29, Thiazolyl Blue Tetrazolium Bromide dye was used. 3-(4,5-dimethylthiazol-2-yl)-2,5-diphenyl tetrazolium bromide (MTT) is a colorimetric assay in which mitochondrial succinate dehydrogenase enzyme converts yellow dye into purple formazan crystals. Cell viability is directly proportional to the intensity of the purple color. Briefly, 3000/well HCT116 and HT29 cells were seeded in 96 well plate. After 24 h of incubation, cells were treated with different concentrations from 0.15 mM to 10 mM of TMA in triplicates for 24 h, 48 h, and 72 h. After incubation, 20 µl MTT dye (5mg/ml) was added to each well and the plate was incubated for 3 hours. Finally, 100 µl of dimethyl sulfoxide (DMSO) was added to dissolve formazan crystals and absorbance was recorded at 570 nm using a Multiskan sky microplate spectrophotometer (Thermo scientific) (27). Untreated cells were taken as control. To calculate % cell viability, the absorbance of untreated cells was noted as 100% viable cells.

### Luciferase assay for ATP determination

Cell viability was also estimated by quantitation of ATP which is an indicator of metabolically active cells. Cellular ATP quantification was performed by CellTiter-Glo^®^ Assay kit as per the manufacturer’s instructions. The CellTiter-Glo^®^ Assay is ideal and accurate for cell viability measurement as it involves a single reagent and is designed for multiwell plate formats and bypasses removal of medium, cell washing, or multiple pipetting steps. Briefly, 5000 cells per well were seeded in 96-well plate for overnight incubation at 37°C. After incubation period, cells were treated with different concentrations of TMA from 0.15 mM to 10 mM for 24 h, 48 h, and 72 h. Thereafter, 100 µl reagent was added to each well and incubated for 10min in dark for signal stabilization. Luminescence was measured at 510 nm using ClarioStar plate reader (BMG Labtech, Ortenberg, Germany). Untreated cells were taken as control. The ATP content of control was considered as 100%.

### Lactate dehydrogenase assay

The lactate to pyruvate process is catalyzed by the LDH enzyme, which is found in the cytosol. When plasma membrane integrity is disrupted, LDH is released into the supernatant, enabling a tetrazolium salt to be converted into a red formazan product. Cell membrane integrity was analyzed using an LDH assay kit according to manufa cturer’s instructions. Cells were seeded into 96-well plate and treated with TMA at 3 different concentrations (2.5 mM, 5 mM, and 10 mM) for 48 h. TMA has no effect below 2.5 mM concentration (data not shown). To estimate the amount of LDH released from cells, 100 µl of supernatant from each well was transferred to a fresh 96-well plate and 100 µl of assay reagent was added followed by 30 min incubation at 37°C. Finally, 20 µl 1N HCl was added as a stop solution. Background absorbance was recorded at 690 nm and subtracted from absorbance recorded at 490 nm (ClarioStar plate reader, BMG Labtech, Ortenberg, Germany). Cells treated with 0.1% triton-100 were considered as positive control or maximum LDH release. Untreated cells were considered as negative control. LDH release from cells was determined using the following formula. 100 × experimental LDH release (OD490)/maximum LDH release (OD490).

### Evaluation of cellular apoptosis

The apoptotic potential of TMA metabolite on cell lines was analyzed by using Annexin V-fluorescein isothiocyanate (FITC)/propidium iodide (PI) double staining kit (ab14085; Abcam, USA). Briefly, 1 × 10^5^cells/ml were cultured in a 6-well plate for 24 h incubation. Afterward, cells were treated with 3 concentrations of TMA (2.5 mM, 5 mM, and 10 mM) for 48 h. Following the treatment period, cells were harvested by trypsinzing and washed twice with PBS. The pellet was resuspended in 500 µl binding buffer with 5 µl PI and 5 µl Annexin V-FITC and kept in dark for 30min. Then, cells were analyzed at 488 nm for apoptosis by flow cytometry (BD Biosciences, FACS Verse, Germany). Cells without treatment were considered as control.

### Superoxide anion radical measurement

HCT116 and HT29 (1×10^5^ cells/well in 6 well plate) cells were treated with TMA. Cells treated with 5 mM and 10 mM TMA for 48 h were harvested by trypsinization and washed with PBS followed by labeling with 5µM dihydroethidium for 30 min in dark at 37°C. Labeled cells were washed with PBS twice. Fluorescence emission of oxidized DHE which corresponds to the amount of superoxide free radicals was measured by flow cytometry (BD Biosciences, FACS Verse, Germany). Untreated cells were considered as a negative control whereas treatment with 250 µM menadione for 30 min were used as a positive control (28).

### Cellular imaging

For brightfield microscope observation, HCT116 cells grown in a 12-well plate (6 × 10^4^/mL) were treated with 3 concentrations (2.5 mM, 5 mM, and 10 mM) of TMA for 24, 48, and 72 h. Untreated cells were considered as control. At the end of the treatment, cells were observed using a nikon microscope equipped with a 20x objective and images were acquired with a digital camera and using the NIS-elements 3.0 software. No. of viable cells were calculated using trypan blue exclusion assay.

### Clonogenic assay

Clonogenic assay or colony formation assay was carried out to determine the effectiveness of cytotoxicity caused by TMA metabolite. Cells were seeded in 6-well plate (1×10^5^ cells/well) and incubated overnight at 37°C in CO_2_ incubator. After incubation, cells were treated with 3 different concentrations of TMA (2.5 mM, 5 mM, and 10 mM) for 48 h. After treatment 400, 800, and 1200 cells were counted from each treatment and grown in a complete medium for 10 days to allow colony formation. Colonies were fixed with 6% glutaraldehyde and stained with 0.5% crystal violet. Colonies with more than 50 cells were counted. Colonies were counted using ImageJ software. The number of colonies of treated cells was compared to that of control samples, and clonogenic efficiency was expressed as the percentage with respect to untreated cells (29).

### Comet assay

DNA damage was calculated *via* comet assay also known as single-cell gel electrophoresis under alkaline conditions (pH>13). Following 48 h treatment of HT29 cells with 3 different concentrations of TMA (2.5 mM, 5mM, 10 mM) TMA for 48 h at 37°C, cells were resuspended in cold PBS, mixed in 0.4% low melting agarose and spread on the frosted glass slide. After the solidification for agarose, the slides were placed in lysis and alkaline buffer for 1 h at 4°C containing 1% Triton X-100, and dimethyl sulfoxide (DMSO), pH 10. Slides were washed with neutralizing buffer for 5 min. Washed slides were kept in electrophoresis buffer for 20 min to relax DNA and then subjected to electrophoresis buffer at 4°C for 40min at an electric field strength of 24 V, 300 mA. After electrophoresis, slides were washed with neutralizing buffer (pH 7.4) for 5 min. DNA was stained with EtBr at a concentration of 0.5mg/ml for 5 min (30). For visualization of DNA damage, images were captured under epifluorescent illumination on a Zeiss microscope. Images were analyzed and the percentage of DNA in head, tail, and tail movement was calculated using NIH ImageJ software.

### Cell cycle distribution

The effect of TMA on cell cycle distribution was studied by exposing both cell lines to 2.5 mM, 5 mM, and 10 mM TMA. Cells exposed to TMA for 48 h were recovered by trypsinization and untreated cells were considered as control. Cells were washed with PBS twice and fixed in 70% ice-chilled ethanol and incubated overnight at 4°C. Incubated cells were washed with cold PBS at 4°C and treated with 35µl 0.1% triton-100 and 50 µl RNase A (10 µg) in 300µl propidium iodide (PI) staining solution followed by incubation in dark for 30 min at 37°C. The staining of incorporated PI was calculated on 10×10^3^ cells with a flow cytometer (BD Biosciences, FACS Verse, Germany) (31, 32). FlowJo v10 (FlowJo, LLC, Ashland, OR, USA) was used for analysis and to quantify the percentage of cells in G0/G1, S and G2/M phases of cell cycle (33).

### Mice study

Female FVB/J mice were purchased from the Jackson Laboratory, United States. The 10 weeks old mice (20-22 g in weight) were randomly divided into 2 experimental groups (TMA given intraperitoneally, TMA given intrarectally) and 2 control groups namely negative control and vehicle control (*n* = 5 per group). The mice in the intrarectal group were given 50 µl of 100 mM TMA intrarectally and mice in the intraperitoneal group were given 50 µl of 100 mM TMA by intraperitoneal injection. The mice in negative control were not given any treatment whereas vehicle control mice were administered intraperitoneally and intrarectally with 50 µl physiological saline. All the experiments were conducted following the ethical code and recommendation issued by the Institutional Biosafety Committee (IBSC) of the National Institute of Immunology, New Delhi.

### Histology

All the mice were euthanized on day 60, dissected and their resected caecum, colon, and rectum length were measured. Histological processing was performed using the Haematoxylin and Eosin staining procedure. To make a firm block, the colon and rectum tissue samples were fixed in 10% neutral formal saline, dehydrated in increasing grades of alcohol, cleaned in xylene, immediately dipped into molten paraffin wax, and lastly embedded in molten paraffin wax. The rotary microtome was then used to slice the hard block enclosing the tissue into 3μm thick sections. The slices were then transferred to a glass slide and stained with hematoxylin and eosin stains. The slides were then examined under a light microscope at ×100 and ×400 magnifications, and images were taken at both magnifications and analyzed for cellular infiltration, ulcers, or any change in cellular morphology. The presence or lack of immune cell infiltration and mucosal presence or absence were used to grade inflammation throughout the colon using a -point scale (score 0 = no change, 1 = mild, 2 = moderate, 3 = severe) (**Table 1**). An Olympus microscope was used for microscopic investigation.

**Table 1:** Histological scoring table: parameters used to score inflammation on a scale from 0 to 3.

Score	0	1	2	3
Infiltration of inflammatory cells	Rare inflammatory cells	Increased number of inflammatory cells	Confluence of inflammatory cells	Transmural extension of inflammatory cell infiltrate
Severity of epithelial damage	Absence of mucosal damage	Lymphoepithelial lesions	Mucosal ulceration	Extensive mucosal damage and extension through deeper structure of intestine wall
Cellular morphology	Normal lining, no crypt distortion	Mild crypt distortion	Moderate crypt distortion	Severe epithelial damage

### Data analysis

The results are expressed as mean value ( ± SEM). Statistical analysis was performed using one-way ANOVA. Differences with *P* value <0.05 were considered statistically significant.

## Results

### Effect of TMA on cellular viability

**Figure 1:**
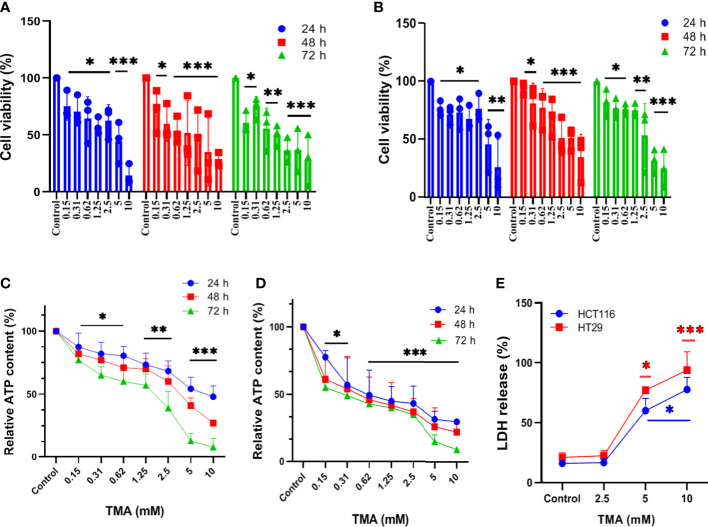
Assessment of cell viability of HCT116 and HT29 exposed with different concentration of TMA. Cytotoxicity of TMA was evaluated by MTT assay (**A**. HCT116, **(B)** HT29), luminescent assay (**C**. HCT116, **D**) HT29) and LDH assay **(E)**. Cells were treated with or without different concentrations of TMA for 24 h, 48 h and 72 h. Cellular viability **(A, B)** and membrane integrity **(E)** were analyzed colorimetrically following treatment with TMA. Below 5 mM TMA exposure, there is no significant increase in LDH release compared to control group. Luminescence was measured for measuring ATP level **(C, D)**. Untreated control is considered as 100%. Difference of average values between untreated and treated cells were tested using one way ANOVA. All tested subjects showed significant decrease in viability at higher concentration of TMA while 10 mM TMA at 72 h proved most cytotoxic to cells. Significance was calculated in comparision to control. The results (mean±SEM) were obtained from 3 independent experiments. Error bars represent standard error from three independent experiments. *indicates significantly different values compared to control group (*P*<0.05)** indicates significantly different values compared to control group (*P*<0.001). ***indicates significantly different values compared to control group (*P*<0.0001).

Cells exposed to different concentrations of TMA (0.15 mM, 0.3 mM, 0.6 mM, 1.2 mM, 2.5 mM, 5 mM, and 10 mM) for 24 h, 48 h, and 72 h showed decreased cell viability as shown in **Figure 1A, B**. TMA inhibited cell viability in a time and dose-dependent manner when compared with control. HCT116 and HT29 cells showed 30% and 45% viability after 24 h exposure to 10 mM TMA respectively whereas after 48 h and 72 h exposure significantly decreased cell viability to approx. 25% and 9% respectively in response to 10 mM TMA. Cell viability was compared with control (without treatment). These results suggest a significant inhibitory effect of TMA in colon carcinoma cells in a time and concentration-dependent manner.

### Effect of TMA on cellular ATP content

TMA decreases cellular viability in both cell lines. To determine the effect of TMA on intracellular ATP levels, we cultured HCT116 and HT29 cell lines in the presence of different concentrations of TMA from 0.15 mM to 10 mM for 24 h, 48 h, and 72 h. As ATP content is not a direct measurement of cell viability but it reflects the metabolic activity of cells, here we confirm that TMA slows down the cellular metabolism in both cell lines. After 24 h exposure of TMA, decrease in ATP content was observed from 0.15 mM to 10 mM TMA and aggravated with time. Cells without TMA were considered as control and their viability was considered 100%. Cell viability was decreased by TMA in a concentration and time dependent manner (**Figure 1C, D**).

### Effect of TMA on membrane integrity

In the TMA-exposed cells, the percentage of LDH release was increased in a dose and time-dependent manner. TMA caused a non-significant release of LDH from cells into the supernatant at concentrations below 5 mM (data not shown). At 5 mM and 10 mM, both HCT116 and HT29 cells released a significant amount of LDH in the supernatant (**Figure 1E**). After 48 hours of TMA exposure, HCT116 exposed to 5 mM and 10 mM TMA released approximately 60% and 80% of total LDH into the supernatant, respectively. Whereas, in the HT29 cell line, 48 hours of exposure caused around 80% of LDH release in 5 mM TMA and 100% of total LDH release in 10 mM TMA. Hence, TMA exposure leads to impaired membrane integrity in both cell lines, with 10 mM being the most hazardous. LDH released from cells treated with 0.1% triton-X100 was considered 100%. Cells with compromised cell membranes cannot maintain the membrane potential necessary for metabolic activity and are considered as dead. Therefore, LDH release is an indirect measure of cellular cytotoxicity and TMA was found to be cytotoxic to both cell lines.

### Effect of TMA on apoptosis

**Figure 2:**
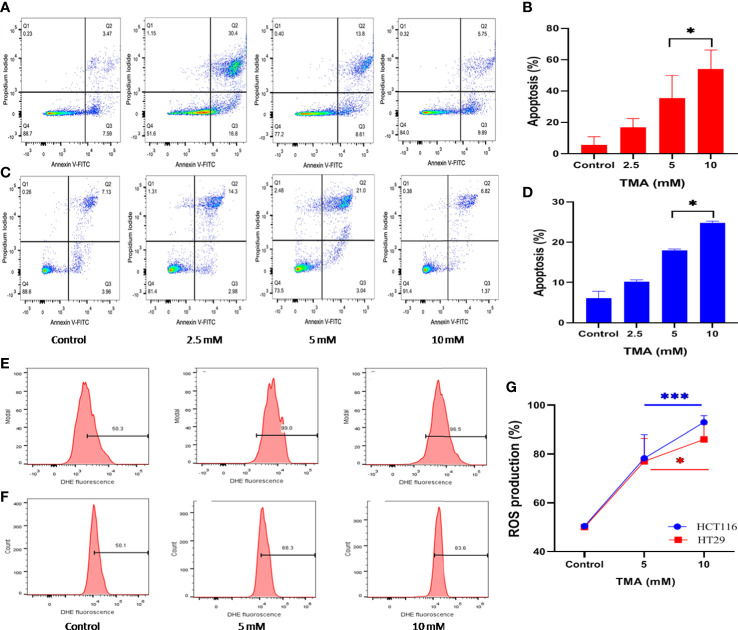
Detection of apoptosis and oxidative stress induced by superoxide radicals. Effect of TMA on HCT116 **(A, E)**, HT29 **(B, F)** cells was checked by flow cytometry. **(A–C)** are representative flow cytometric dot plot showing the percentage of viable cells (annexin V-FITC-, PI-), early apoptotic cells (annexin V-FITC+, PI-), late apoptotic cells (annexin V-FITC+, PI+) and necrotic cells (annexin V-FITC-, PI+). Results are plotted as no. of apoptotic cells (annexin V-FITC+, PI-) which shows significant increase in apoptosis with increase in concentration of TMA **(B, D)**. Negative control cells were treated with solvent only. TMA induces production of anion superoxide radical in both cell lines, **(E)**. HCT116 and **(F)**. HT29. HCT116 and HT29 cells were treated with 5 mM and 10 mM TMA for 48 h. Difference of average values between untreated and treated cells were tested using paired student’s t-test. Both cell lines showed significant increase in apoptosis and ROS production particularly 10 mM TMA proved most toxic to cells. The exposure of TMA led to an increase in fluorescence intensity in both cell lines thereby, shifting histogram towards right **(G)**. The results (mean±SEM) were obtained from 3 independent experiments. *indicates significantly different values compared to control group (*P*<0.05). ***indicates significantly different values compared to control group (*P*<0.0001).

Annexin V and PI labeled cells were checked for the apoptotic effects of TMA by flow cytometry and data demonstrated that TMA induces apoptosis in both cell lines. At doses below 2.5 mM, TMA caused a non-significant increase in apoptosis (data not shown). PI stains cells exclusively in late apoptosis or necrosis, whereas Annexin V may be found in both early and late phases of apoptosis. Early apoptotic cells (lower right quadrant) were stained for annexin V but not for PI, whereas late apoptotic cells were positive for both annexin V and PI (upper right quadrant). Apoptosis was analyzed by counting both early and advanced apoptotic cells (**Figure 2A, C**). As shown in **Figure 2B**, HCT116 cells were treated with three different doses of TMA for 48 hours: 2.5 mM, 5 mM, and 10 mM, when compared to the control (10%), the percentage of apoptosis rose to around 15%, 22%, and 47%, respectively. However, after the 48 hour exposure to TMA, the proportion of apoptotic HT29 cells rose to about 11%, 17%, and 24%, respectively, as compared to the control (8%) (**Figure 2D**). This data suggest that TMA increases apoptosis rate by inducing cytotoxicity as discussed in above sections.

### ROS production induced by TMA

TMA treated cells showed an increased production of superoxide as detected by higher DHE fluorescence when compared to untreated cells (**Figure 2E, F**). TMA causes 56 ± 9.66% and 86 ± 2.7% increase in ROS production in 5 mM and 10 mM exposed HCT116 cells respectively. Whereas, the HT29 cells showed 55 ± 9.3% and 73.2 ± 6.1% increase in ROS production after 48 h of TMA exposure (**Figure 2G**). Superoxide detection was not statistically significant for TMA concentrations less than 5 mM. Increased ROS production is the hallmark of inflammation which can lead to oxidative stress. Oxidative stress is a known threat to DNA integrity. In case of any genomic damage, cells try to repair through DNA damage repair pathways but if the cell fails to repair it undergoes apoptosis. The results are in correlation with comet assay, imaging, cell viability and apoptosis results as at high concentrations of TMA such as 5 mM and 10 mM, ROS cause DNA damage, and cells undergo apoptosis.

### Cellular imaging

**Figure 3:**
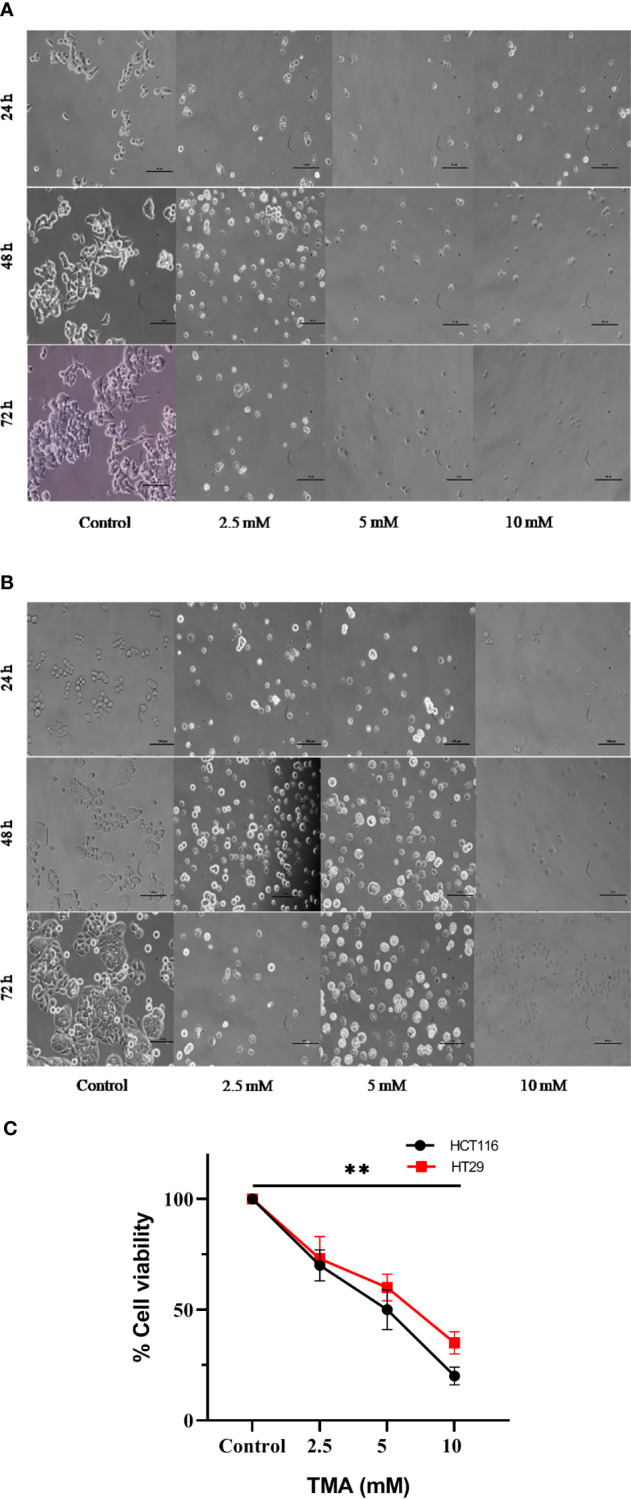
Bright field images of HCT116 **(A)** and HT29 **(B)** showing changes in cellular morphology, detachment from surface, decreased no. with increase in TMA concentration and time of exposure. Decrease in cell viability was observed after exposure **(C)**. ** indicates significant decrease in cell viability compared to control group (P<0.001).

Under an inverted microscope, the TMA-induced morphological alterations in HCT116 and HT29 cells were evaluated and found that cells at 2.5 mM, 5 mM, and 10 mM doses for 48 h displayed obvious indications of morphological alterations, including rounding and scathing of cells with disorganized cell layers, as compared to control untreated cells (**Figure 3A, B**). Additionally, the cells got separated from one another and floated freely in the media. All these changes induced by TMA, such as losing shape, looking lobulated, rupture of plasma membrane, changes in architecture are considered hallmark of death. A decrease in no. of viable cells was found by trypan blue exlusion assay (**Figure 3C**). The results of cellular imaging are in correlation with cytotoxicity assays; MTT, ATP, and LDH assay.

### Cytotoxic effect of TMA on colony forming ability

**Figure 4:**
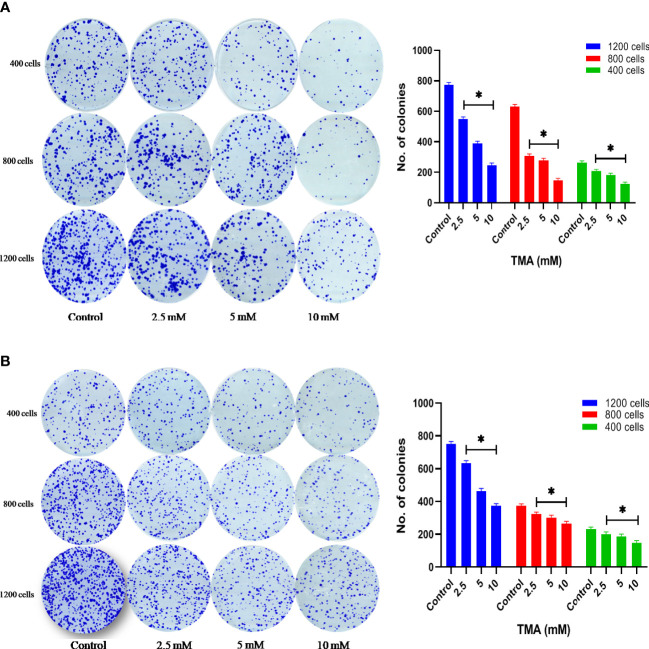
Clonogenic assay. TMA inhibits colony formation of **(A)** HCT116 and **(B)** HT29 cells. Evaluation of colony forming ability in HCT116 and HT29 cell line exposed to various concentrations of TMA for 48 h. TMA inhibits the colony forming ability of HCT116 in a concentration dependent manner. Results were plotted as no. of colonies formed after 14 days (shown right to the colony images). Negative control cells were treated with cell culture water only. Difference of average values between untreated and treated cells were tested using paired student’s t-test. All tested subjects showed significant decrease in viability while 10 mM TMA proved most cytotoxic to cells. The results (mean±SEM) were obtained from 3 independent experiments. *indicates significantly different values compared to control group (*P*<0.05).

HCT116 and HT29 cells can proliferate and form colonies. The effect of TMA on the colony-forming ability of cells was evaluated by performing clonogenic assay which helps in determining the degree of inhibition of colony formation and offers insight into the potential post-exposure behavior of cells. To elucidate the post exposure effect of TMA, we exposed HCT116 and HT29 cells which are known to form colonies with 5 different concentrations of TMA (2.5 mM, 5 mM, and 10 mM) for 48 h and the no. of colonies formed were plotted. As is evident from the representative images in **Figures 4A, B**, TMA inhibited cell growth in a concentration-dependent manner after exposure. Relative to control (considered as 100% colony formation) both HCT116 and HT29 cells exposed with 2.5 mM, 5 mM, and 10 mM showed approximately 35%, 32%, and 21% colony formation, respectively. Treatment of 10mM TMA led to most significant decrease in the growth of cells. This demonstrates that TMA is efficient in preventing colony formation and highlights its potential effectiveness as a toxin. The result of clonogenic assay was in correlation with other cytotoxicity measurement assays such as MTT and LDH assay.

### Genotoxic effect of TMA

**Figure 5:**
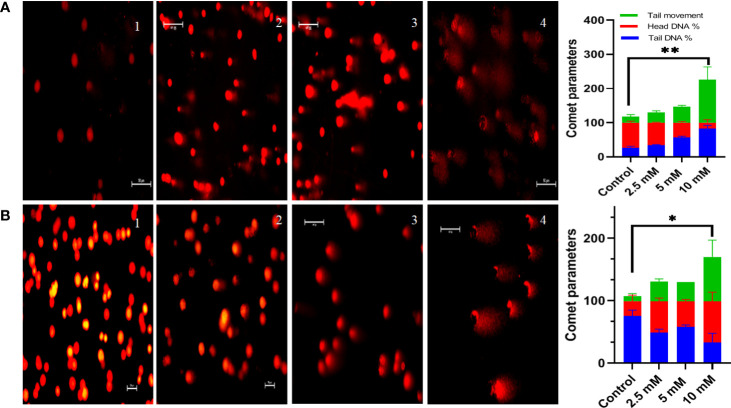
Comet assay. Comet assay was performed to evaluate genotoxicity induced by TMA. Cells were treated with different concentrations of TMA for 48 h. Representative images at X200 magnification shows that TMA causes DNA damage in dose dependent manner as shown in **(A)** HCT116 cells treated with and without TMA and **(B)** HT29 cells treated with and without TMA. 1, 2, 3, and 4 represent control, 2.5 mM, 5 mM and 10 mM treatment respectively. DNA damage was enumerated using the OpenComet software tool in imageJ software (https://imagej.net/software/fiji/ ) as shown in **(C)** HCT116 and **(D)** HT29 respectively. Results were plotted as comet parameters. Both cell lines showed significant DNA damage at 10 mM TMA. The results (mean±SEM) were obtained from 3 independent experiments. *indicates significantly different values compared to control group (*P*<0.05) **indicates significantly different values compared to control group (*P*<0.001).

Comet assay was used to measure DNA damage. Cells exposed to TMA for 48 h exhibited significant DNA damage at concentrations of more than 2.5 mM. The comet assay reveals DNA fragmentation in both HCT116 and HT29 cell lines at 2.5 mM, 5 mM, and 10 mM. In response to TMA, cells collected damaged DNA that migrated out of the cell when an electric field was applied, which was qualitatively evaluated using the Image J software based on the intensity of the comet tail produced. With the increase in concentration of TMA, the % of DNA in head decreases from 74% (control) to 3% (10 mM TMA), and % of DNA increases in tail from 25% (control) to 96% (10 mM TMA) whereas the tail movement increases from 6% (control) to 179% (10 mM TMA) (**Figure 5**). Significant DNA damage observed in TMA exposed cells strengthens the finding obtained through cell viability studies suggesting that DNA damage leads to cell death.

### G2-M phase cell cycle arrest by TMA

**Figure 6:**
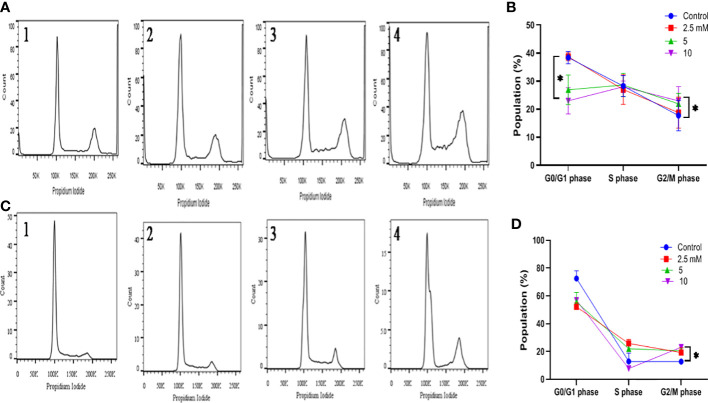
Effect of TMA on cell cycle progression. Evaluation of cell cycle HCT116 **(A)** and HT29 **(B)** cells were treated with various concentrations of TMA for 48 h. Untreated cells were considered as negative control. **(A)** and **(B)** showed the quantification of cell cycle distribution. TMA induced cell cycle arrest. 1, 2, 3, and 4 represent control, 1.25 mM, 5 mM and 10 mM treatment respectively. The distribution of various phase of HCT116 and HT29 cells are represented in **(C, D)** Difference of average values between untreated and treated cells were tested using one-way ANOVA. The results (mean±SEM) were obtained from 3 independent experiments. *indicates significantly different values compared to control group (*P*<0.05).

The alterations in cell cycle were examined to determine the causes for the decrease in cell number following exposure to TMA. After 48 hours of incubation with TMA, the G2 phase cell distribution increased, but the S phase cell distribution did not alter significantly. In general, programmed cell death is linked to cell cycle arrest. After DNA replication, the cell enters the G2 phase, which is a time of protein synthesis and fast cell growth that prepares the cell for mitosis. The G2 phase was halted, it affected cell division and, as a result, hampered the entire life process for HCT116 and HT29 cells. We can conclude that there was a statistically significant increase in phase G2/M following the treatment with 5mM and 10mM TMA from 17.76 ± 5.41 to 23.96 ± 5.08 in HCT116 and from 12.8 ± 1.74 to 23.1 ± 0.98 in HT29 cells (**Figure 6**). Both tested subjects showed significant cell cycle arrest at G2/GM checkpoint of cell cycle while most effective at 10mM TMA at 48 h. With the onset of these events, the shape of the cells began to change from enlargement to spherical transformation, and the vesicle structure began to expand. Interestingly, the G2 phase was blocked, which had an impact on the division of cells, thereby affecting the whole process of life for HCT116 and HT29 cells. With the occurrence of these phenomena, the shape of the cells to spherical transformation, vesicle structure also began to increase leading to the death of cell. These results suggest that TMA has a toxic effect against HCT116 and HT29 cells, possibly *via* the halting cell cycle leading to induction of apoptosis.

### TMA induces inflammation in mice

**Figure 7:**
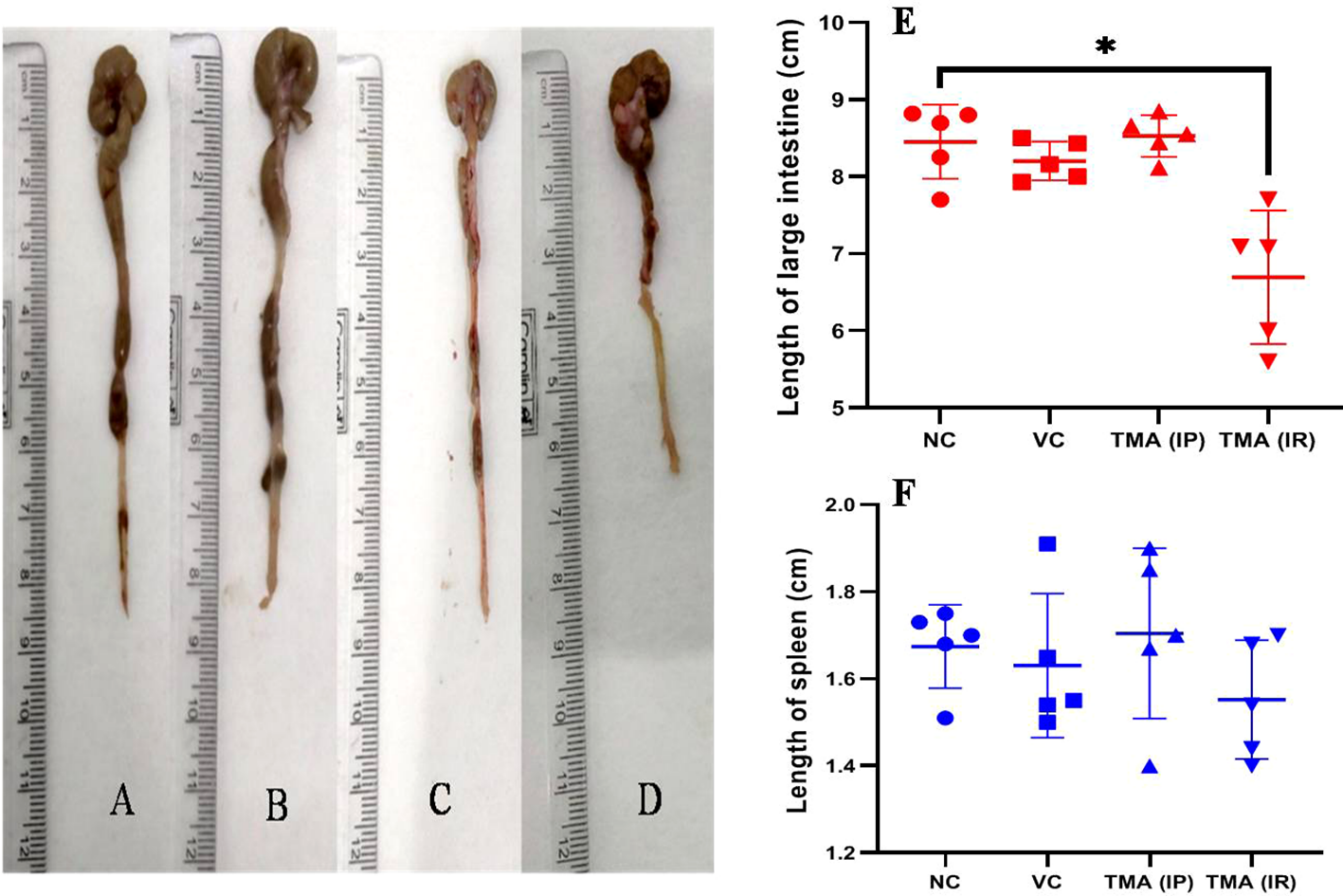
Length of large intestine of mice of Negative control **(A)**, Vehicle control **(B)**, TMA given intraperitoneally **(C)** and TMA given intrarectally **(D)**. Graph showing the length of intestine and spleen in different groups **(E, F)** respectively. *p<0.05.

**Figure 8:**
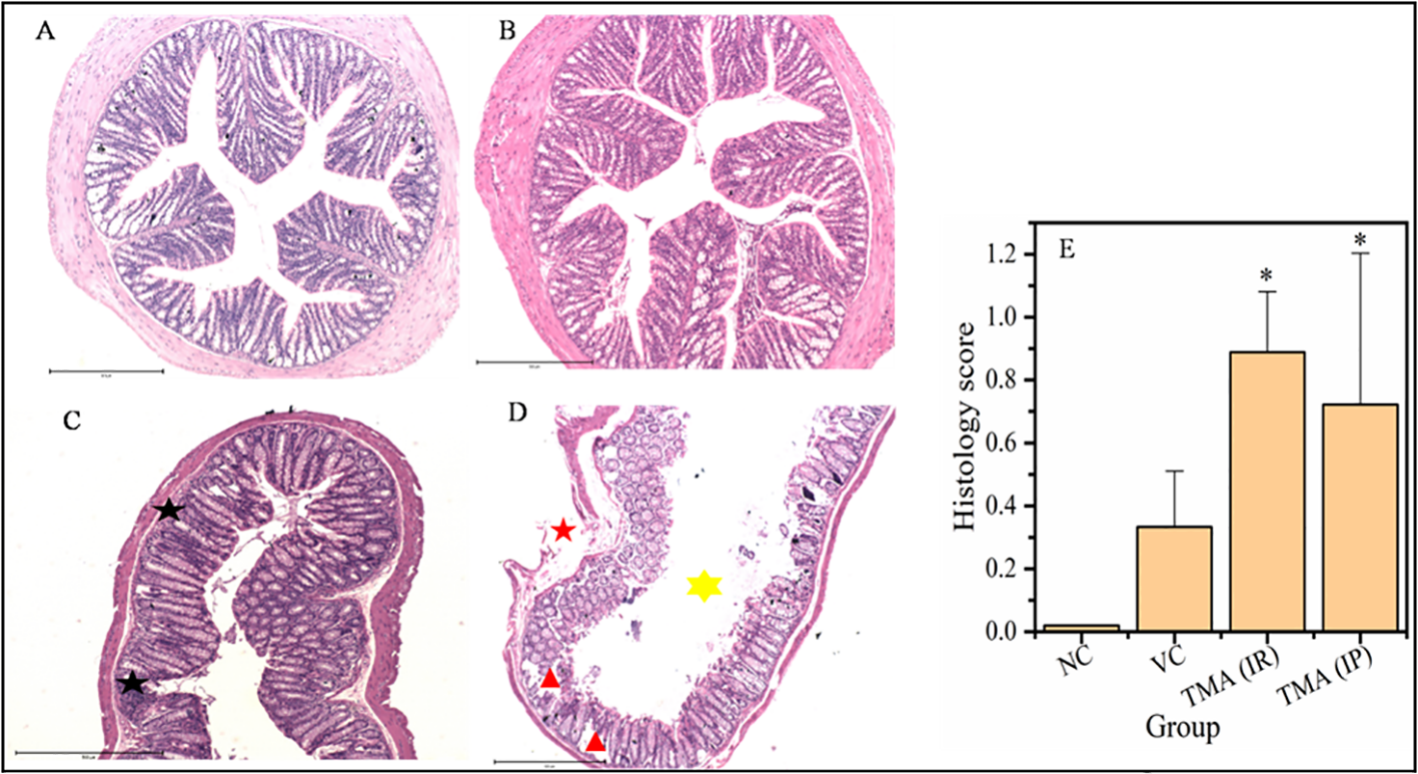
Micrographs of the colon **(A-D)** and histological score of all groups **(E)**. H & E sections of colon from mice exposed to TMA intraperitoneally **(C)** and intrarectally **(D)** or negative control **(A)** or vehicle control **(B)** are shown. Severe inflammation characterized by severe cellular infiltration persisted in the colon of mice exposed to TMA intrarectally **(D)** compared to negative and vehicle control. Black star point to cellular infiltration, red star points to damaged muscularis, yellow star points to increased lumen and red triangle points to distorted crypts. The histology scores for the inflammation of these groups are summarized in **(E)**. The histological score showed a significant increase in inflammation after TMA exposure. *p<0.05.

**Figure 9:**
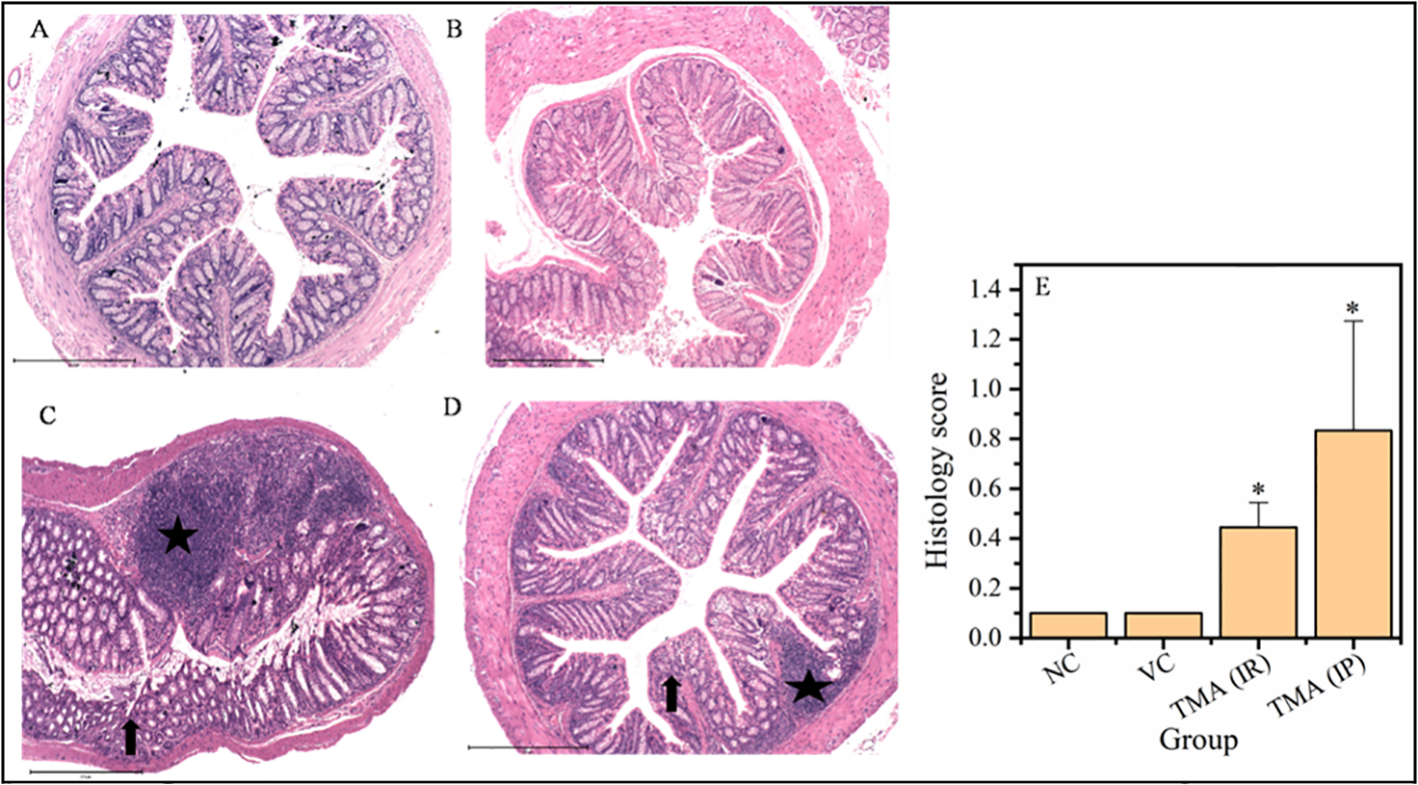
Micrographs of the rectum **(A-D)** and histological score of all groups **(E)**. H & E sections of rectum from mice exposed to TMA intraperitoneally (IP) **(C)** and intrarectally (IR) **(D)** or negative control **(A)** or vehicle control **(B)** are shown. Severe inflammation characterized by severe cellular infiltration persisted in the colon of mice exposed to TMA intrarectally **(D)** compared to negative and vehicle control. The histology scores for the inflammation of these groups are summarized in **(E)**. Black star points to cellular infiltration and black arrow represents crypt distortion in TMA exposed mice rectum. The histological score showed a significant increase in inflammation after TMA exposure. *p<0.05.

TMA reflects the deleterious effects as shown in the cell line study; therefore, *in vivo* effect was studied in mice model. FVB/J mice were injected with TMA for 4 weeks. A series of signs of inflammation and/or ulceration were monitored in all the mice groups. Additionally, the weight, stool consistency, and rectal bleeding were monitored. Compared to control, the TMA exposed mice showed significant alteration in normal colon and rectum tissue. The mean body weight of mice was not significantly affected when compared to negative and vehicle control. On day 60, an examination of the length and histology of the experimental mice’s colons and rectum was performed. Macroscopic examination of the intestine demonstrated that TMA administration significantly reduced the length of the colon (**Figures 7A–E**). No significant increase in spleen length was found among the negative, vehicle and experimental groups (**Figure 7F**). Samples of large intestine from animals were evaluated from hematoxylin/eosin (H&E)-stained sections. Notably, H&E staining indicated a marked increase in inflammatory cell infiltration in the both colon and rectum of TMA group mice. However, the TMA-intrarectal group presented more histological injury in the rectum, with lesser denudation and ulceration of colon epithelial cells as compared to rectum. However, TMA, given intraperitoneally, induces infiltration of immune cells more in the colon region than rectum (**Figure 8**). The histological scores further confirmed that intrarectal and intraperitoneal TMA administration might increase the extent of ulceration and inflammation (**Figure 9**). The route of administration of TMA determines the extent of inflammation in intestine as TMA given by intraperitoneal (IP) route induced less inflammation than that of intrarectal administration. The difference may be because more amount of TMA comes in direct contact to intestinal epithelium in intrarectal administration.

## Discussion

Present work on human adenocarcinoma cell lines, HCT116 and HT29, and mice study show that acute as well as longer-term exposure to TMA affects proliferation, and morphological characteristics of cells. Cell lines were tested with 10 different concentrations of TMA for short (24 h) and longer-term (72 h) exposure. Both cell lines were found to have decreased proliferation rate in response to treatment with various concentrations of TMA within 1 day and the effect worsen with increased time of exposure such as decreased proliferation, cell cycle arrest, DNA damage leading to cell death. Proliferation may be reduced as a result of an adaptive response to diminished ATP production (34). Indeed, a reduction in ATP level is linked to cell death and is chosen as an indication. It is also used as a proxy to indicate cellular metabolic activity (35). However, accumulation of cells in G2/M phase was reported at an exposure of high concentration of TMA which might be the consequence of DNA damage leading to apoptosis. The G2/M arrest gives the cell extra time to repair its DNA (36–38). Cell undergoes apoptosis if DNA repair fails (39). Apoptosis is a subsequent response to DNA damage that helps the organism protect itself from a damaged cell. Our data show that there is dose-dependent induction of DNA damage by TMA in both cell lines as reported by comet assay. Increased reactive oxygen species production is also observed at higher exposure which is often correlated with DNA damage. In point of fact, both cell lines showed no sign of apoptosis when exposed to acute (<24 h) term at a lower concentration of TMA. After 48 hours of treatment with TMA, HCT116 and HT29 cells produced more anion superoxide, demonstrating that there is a lag period for cellular response to the treatment. Anion superoxide is formed primarily in the mitochondrial complexes I and III during oxidative phosphorylation in most eukaryotic cell types, especially when mitochondrial complex activity is inhibited (40–46). Our data shows the dose-dependent effect of TMA on cellular morphology which can be correlated with typical morphological features of apoptosis such as shrinkage of cells and membrane blebbing. Cells exposed to high concentrations of TMA such as 5 mM and 10 mM have significantly decreased survival rates which reflects the extent of damage to DNA and other cellular organelles.

TMA has been suggested as a uremic toxin and correlated with cardiovascular disease as well (16). Furthermore, the goal of our research is to check if this bacterial metabolite is genotoxic to colonic cells. Because those cells face the large intestine lumen, where TMA is formed, they are likely the cells in the body that are most accessible to this bacterial product. TMA appears to be a metabolic threat as well as a genotoxic luminal bacterial metabolite targeting colonic epithelial cells, based on our findings. With these considerations in mind, the findings of this study suggest that when TMA is present in excess, the colon epithelial layer is exposed to a luminal content defined by the presence of toxic compounds, including TMA. Taking the results into consideration, our study shows that TMA is both a genotoxic and cytotoxic agent which can be a metabolic troublemaker in human colonocytes.

Animal study has demonstrated the deleterious effects of TMA in the intestine. In both colon and rectum, with the exposure of TMA by both routes (intrarectal and intraperitoneal), inflammatory cells invasion and necrotic area increased. In the intrarectal group, rectal region was more affected whereas, in the intraperitoneal group, colon region was affected more with TMA. When compared to negative and vehicle control mice groups, TMA exposed groups had higher colonic and rectal epithelial damage as characterized by neutrophil infiltration and increased histological score. Therefore, TMA induces inflammation in mice was verified. As TMA is produced by gut microbiota, any alteration in gut microbiota especially the enrichment of Gammaproteobacteria, may lead to increased production of TMA in gut (47). The increased exposure of gut epithelium to TMA may induce inflammation which can lead to the condition known as oxidative stress. As we showed in cell line experiments, prolonged oxidative stress makes the DNA unstable leading to development of severe intestinal disorders. The serosal abrasion, crypt distortion and denudation indicate the deleterious effects of TMA. The mechanisms involved in TMA induced inflammation are still not resolved, nor how TMA regulates inflammation, influences metabolism and other biological activities in large intestine. Finally, our finding contributes to our understanding of the impact of bacterial metabolites *in vivo* and *in vitro*. We show that TMA decreases cell viability and ATP content of cells in acute exposure. Additionally, it leads to compromised membrane integrity, cell cycle arrest and apoptosis in chronic exposure. It causes severe inflammation, damage to the epithelial lining of mice, shortening the large intestine strengthening the view that TMA is deleterious and might be a contributing factor in microbiota-induced intestinal diseases. Future studies are needed to understand the mechanistic pathway of TMA induced inflammation and how to specifically modulate the microbiota to control the TMA production and thus predict possible therapy with personalized strategies in intestinal inflammation.

## Data availability statement

The datasets presented in this study can be found in online repositories. The names of the repository/repositories and accession number(s) can be found in the article/supplementary material.

## Ethics statement

The animal study was reviewed and approved by Institutional Animal Ethics Committee (IAEC) of National Institute of Immunology (Permit IAEC # 492/18).

## Author contributions

AK conceived and designed the research. RJ conducted the experiments. AK, GM, and MS contributed new reagents or analytical tools. RJ and AK analyzed the data. RJ wrote the manuscript. All authors contributed to the article and approved the submitted version.

## References

[1] Simó C García-Cañas V. Dietary bioactive ingredients to modulate the gut microbiota-derived metabolite TMAO. new opportunities for functional food development. Food Funct (2020) 11:6745–76. doi: 10.1039/D0FO01237H 32686802

[2] Rath S Heidrich B Pieper DH Vital M. Uncovering the trimethylamine-producing bacteria of the human gut microbiota. Microbiome (2017) 5:54. doi: 10.1186/s40168-017-0271-9 28506279 PMC5433236

[3] Janeiro MH Ramírez MJ Milagro FI Martínez JA Solas M. Implication of trimethylamine n-oxide (TMAO) in disease: Potential biomarker or new therapeutic target. Nutrients (2018) 10:1398. doi: 10.3390/nu10101398 30275434 PMC6213249

[4] Gatarek P Kaluzna-Czaplinska J. Trimethylamine n-oxide (TMAO) in human health. EXCLI J (2021) 20:301–19. doi: 10.17179/excli2020-3239 PMC7975634 33746664

[5] Fennema D Phillips IR Shephard EA. Trimethylamine and trimethylamine n-oxide, a flavin-containing monooxygenase 3 (FMO3)-mediated host-microbiome metabolic axis implicated in health and disease. Drug Metab Dispos (2016) 44:1839–50. doi: 10.1124/dmd.116.070615 PMC5074467 27190056

[6] Teft WA Morse BL Leake BF Wilson A Mansell SE Hegele RA. Identification and characterization of trimethylamine-n-oxide uptake and efflux transporters. Mol Pharm (2017) 14:310–8. doi: 10.1021/acs.molpharmaceut.6b00937 PMC6580852 27977217

[7] Borrel G McCann A Deane J Neto MC Lynch DB Brugère J-F. Genomics and metagenomics of trimethylamine-utilizing archaea in the human gut microbiome. ISME J (2017) 11:2059–74. doi: 10.1038/ismej.2017.72 PMC5563959 28585938

[8] Fiori J Turroni S Candela M Brigidi P Gotti R. Simultaneous HS-SPME GC-MS determination of short chain fatty acids, trimethylamine and trimethylamine n-oxide for gut microbiota metabolic profile. Talanta (2018) 189:573–8. doi: 10.1016/j.talanta.2018.07.051 30086962

[9] Zeisel SH Warrier M. Trimethylamine n-oxide, the microbiome, and heart and kidney disease. Annu Rev Nutr (2017) 37:157–81. doi: 10.1146/annurev-nutr-071816-064732 28715991

[10] Hoyles L Jiménez-Pranteda ML Chilloux J Brial F Myridakis A Aranias T. Metabolic retroconversion of trimethylamine n-oxide and the gut microbiota. Microbiome (2018) 6:73. doi: 10.1186/s40168-018-0461-0 29678198 PMC5909246

[11] Yu Z-L Zhang L-Y Jiang X-M Xue C-H Chi N Zhang T-T. Effects of dietary choline, betaine, and l-carnitine on the generation of trimethylamine-n-oxide in healthy mice. J Food Sci (2020) 85:2207–15. doi: 10.1111/1750-3841.15186 32572979

[12] Arias N Arboleya S Allison J Kaliszewska A Higarza SG Gueimonde M. The relationship between choline bioavailability from diet, intestinal microbiota composition, and its modulation of human diseases. Nutrients (2020) 12:2340. doi: 10.3390/nu12082340 32764281 PMC7468957

[13] Obeid R Awwad HM Rabagny Y Graeber S Herrmann W Geisel J. Plasma trimethylamine n-oxide concentration is associated with choline, phospholipids, and methyl metabolism. Am J Clin Nutr (2016) 103:703–11. doi: 10.3945/ajcn.115.121269 26864355

[14] Cheng Y Xie G Chen T Qiu Y Zou X Zheng M. Distinct urinary metabolic profile of human colorectal cancer. J Proteome Res (2012) 11:1354–63. doi: 10.1021/pr201001a 22148915

[15] Lewin L. Lehrbuch der toxikologie: für Ärzte, studirende und apotheker. In: Urban & schwarzenberg (Wien: Urban & Schwarzenberg) (1885). p. 476.

[16] Jaworska K Hering D Mosieniak G Bielak-Zmijewska A Pilz M Konwerski M. TMA, a forgotten uremic toxin, but not TMAO, is involved in cardiovascular pathology. Toxins (2019) 11:490. doi: 10.3390/toxins11090490 31454905 PMC6784008

[17] Ufnal M. Trimethylamine, a toxic precursor of trimethylamine oxide, lost in medical databases. J Nutr (2020) 150:419. doi: 10.1093/jn/nxz265 32030429

[18] Messenger J Clark S Massick S Bechtel M. A review of trimethylaminuria: (Fish odor syndrome). J Clin Aesthet Dermatol (2013) 6:45–8. PMC3848652 24307925

[19] Lee S-K Kim D-H Jin C-B Yoo H-H. Determination of urinary trimethylamine and trimethylamine n-oxide by liquid chromatography-tandem mass spectrometry using mixed-mode stationary phases. Bull Korean Chem Soc (2010) 31:483–6. doi: 10.5012/bkcs.2010.31.02.483

[20] D’Angelo R Scimone C Esposito T Bruschetta D Rinaldi C Ruggeri A. Fish odor syndrome (trimethylaminuria) supporting the possible FMO3 down expression in childhood: a case report. J Med Case Rep (2014) 8:328. doi: 10.1186/1752-1947-8-328 25288227 PMC4190592

[21] Bain MA Faull R Fornasini G Milne RW Evans AM. Accumulation of trimethylamine and trimethylamine-n-oxide in end-stage renal disease patients undergoing haemodialysis. Nephrol Dial Transplant (2006) 21:1300–4. doi: 10.1093/ndt/gfk056 16401621

[22] Neyer P Bernasconi L Fuchs JA Allenspach MD Steuer C. Derivatization-free determination of short-chain volatile amines in human plasma and urine by headspace gas chromatography-mass spectrometry. J Clin Lab Anal (2020) 34:e23062. doi: 10.1002/jcla.23062 31595561 PMC7031570

[23] Yang S Li X Yang F Zhao R Pan X Liang J. Gut microbiota-dependent marker TMAO in promoting cardiovascular disease: Inflammation mechanism, clinical prognostic, and potential as a therapeutic target. Front Pharmacol (2019) 10:1360. doi: 10.3389/fphar.2019.01360 31803054 PMC6877687

[24] Bordoni L Samulak JJ Sawicka AK Pelikant-Malecka I Radulska A Lewicki L. Trimethylamine n-oxide and the reverse cholesterol transport in cardiovascular disease: a cross-sectional study. Sci Rep (2020) 10:18675. doi: 10.1038/s41598-020-75633-1 33122777 PMC7596051

[25] Jalandra R Dalal N Yadav AK Verma D Sharma M Singh R. Emerging role of trimethylamine-n-oxide (TMAO) in colorectal cancer. Appl Microbiol Biotechnol (2021) 105:7651–60. doi: 10.1007/s00253-021-11582-7 34568962

[26] Mackay RJ McEntyre CJ Henderson C Lever M George PM. Trimethylaminuria: Causes and diagnosis of a socially distressing condition. Clin Biochem Rev (2011) 32:33–43. 21451776 PMC3052392

[27] Kumar P Nagarajan A Uchil PD. Analysis of cell viability by the MTT assay. Cold Spring Harb Protoc (2018) 2018):pdb.prot095505. doi: 10.1101/pdb.prot095505 29858338

[28] Andriamihaja M Lan A Beaumont M Audebert M Wong X Yamada K. The deleterious metabolic and genotoxic effects of the bacterial metabolite p-cresol on colonic epithelial cells. Free Radic Biol Med (2015) 85:219–27. doi: 10.1016/j.freeradbiomed.2015.04.004 25881551

[29] Franken NAP Rodermond HM Stap J Haveman J van Bree C. Clonogenic assay of cells. vitro Nat Protoc (2006) 1:2315–9. doi: 10.1038/nprot.2006.339 17406473

[30] Rojas E Lopez MC Valverde M. Single cell gel electrophoresis assay: methodology and applications. J Chromatogr B: Biomed Sci Appl (1999) 722:225–54. doi: 10.1016/S0378-4347(98)00313-2 10068143

[31] Pozarowski P Darzynkiewicz Z. Analysis of cell cycle by flow cytometry. In: Schönthal AH, editor. Checkpoint controls and cancer: Volume 2: Activation and regulation protocols. methods in molecular biology. Totowa, NJ: Humana Press (2004). p. 301–11. doi: 10.1385/1-59259-811-0:301 15220539

[32] Darzynkiewicz Z Juan G Bedner E. Determining cell cycle stages by flow cytometry. Curr Protoc Cell Biol (1999) 1:8.4.1–8.4.18. doi: 10.1002/0471143030.cb0804s01 18228389

[33] McCoy JP. Flow cytometry software. In: McManus LM Mitchell RN, editors. Pathobiology of human disease. San Diego: Academic Press (2014). p. 3664–77. doi: 10.1016/B978-0-12-386456-7.07103-3

[34] Leschelle X Goubern M Andriamihaja M Blottière HM Couplan E Gonzalez-Barroso M-M. Adaptative metabolic response of human colonic epithelial cells to the adverse effects of the luminal compound sulfide. Biochim Biophys Acta (BBA) - Gen Subj (2005) 1725:201–12. doi: 10.1016/j.bbagen.2005.06.002 15996823

[35] Braissant O Astasov-Frauenhoffer M Waltimo T Bonkat G. A review of methods to determine viability, vitality, and metabolic rates in microbiology. Front Microbiol (2020) 11:547458. doi: 10.3389/fmicb.2020.547458 33281753 PMC7705206

[36] Dhanalakshmi S Agarwal P Glode LM Agarwal R. Silibinin strongly synergizes human prostate carcinoma DU145 cells to doxorubicin-induced growth inhibition, G2-m arrest, and apoptosis. Clin Cancer Res 8: 3512–3519 2002 Clin Cancer Res (2002) 8:3311–4. doi: 10.1002/ijc.11299 12429642

[37] Ventura E Giordano A. Cell cycle. In: Reference module in life sciences. Elsevier (2019). doi: 10.1016/B978-0-12-809633-8.90189-4

[38] Stark GR Taylor WR. Analyzing the G2/M checkpoint. Methods Mol Biol (2004) 280:51–82. doi: 10.1385/1-59259-788-2:051 15187249

[39] Borges HL Linden R Wang JY. DNA Damage-induced cell death. Cell Res (2008) 18:17–26. doi: 10.1038/cr.2007.110 18087290 PMC2626635

[40] Cadenas E Davies KJA. Mitochondrial free radical generation, oxidative stress, and aging11This article is dedicated to the memory of our dear friend, colleague, and mentor Lars ernster (1920–1998), in gratitude for all he gave to us. Free Radical Biol Med (2000) 29:222–30. doi: 10.1016/S0891-5849(00)00317-8 11035250

[41] Dupré-Crochet S Erard M Nüβe O. ROS production in phagocytes: why, when, and where? J Leukocyte Biol (2013) 94:657–70. doi: 10.1189/jlb.1012544 23610146

[42] Figueira TR Barros MH Camargo AA Castilho RF Ferreira JCB Kowaltowski AJ. Mitochondria as a source of reactive oxygen and nitrogen species: From molecular mechanisms to human health. Antioxid Redox Signaling (2013) 18:2029–74. doi: 10.1089/ars.2012.4729 23244576

[43] Kamizato M Nishida K Masuda K Takeo K Yamamoto Y Kawai T. Interleukin 10 inhibits interferon γ- and tumor necrosis factor α-stimulated activation of NADPH oxidase 1 in human colonic epithelial cells and the mouse colon. J Gastroenterol (2009) 44:1172. doi: 10.1007/s00535-009-0119-6 19714290

[44] Raha S Robinson BH. Mitochondria, oxygen free radicals, disease and ageing. Trends Biochem Sci (2000) 25:502–8. doi: 10.1016/S0968-0004(00)01674-1 11050436

[45] Turrens JF. Superoxide production by the mitochondrial respiratory chain. Biosci Rep (1997) 17:3–8. doi: 10.1023/A:1027374931887 9171915

[46] Venditti P Di Stefano L Di Meo S. Mitochondrial metabolism of reactive oxygen species. Mitochondrion (2013) 13:71–82. doi: 10.1016/j.mito.2013.01.008 23376030

[47] Macpherson ME Hov JR Ueland T Dahl TB Kummen M Otterdal K. Gut microbiota-dependent trimethylamine n-oxide associates with inflammation in common variable immunodeficiency(2020) (Accessed December 17, 2022). 10.3389/fimmu.2020.574500 PMC7525000 33042155

